# Cases of Poisoning by Opium

**Published:** 1823-02

**Authors:** J. C. Yeatman

**Affiliations:** Surgeon Extraordinary to his Royal Highness the Duke of Gloucester, and Member of the Royal College of Surgeons.


Art. VIII.—*Cases of Poisoning by Opium.* By J. C. Yeatman, Esq. Surgeon Extraordinary to his Royal Highness the Duke of Gloucester, and Member of the Royal College of Surgeons.

In addition to former contributions of the above kind, and with a view to increase the common stock of such cases, it may be useful to state the following. Cases of poisoning by opium are by no means frequently met with in the records of medicine; and therefore it is the more praiseworthy of the Editors of this long-established Journal, (a work which, with its General Index, now forms an excellent medical library,) to give publicity, particularly as they have done in their Number for September, to all such cases as come to their knowledge.

Case I.—A young man, at Shepton Mallet, in this county, swallowed two ounces of laudanum. A surgeon was immediately sent for to the patient, who administered nearly twenty grains of tartrate of antimony and elaterium, with a large portion of citric acid. This remedy acted instantly on the stomach and bowels, and the patient was recovered from the narcotic influence of the poison.

Case II.—Mr. J. W., æt. sixty-five, of robust constitution, addicted to dram and beer drinking, and to the smoking and chewing of tobacco, swallowed, in a state of intoxication, nearly two ounces of laudanum. I saw him in bed, within half an hour after the drug had been in the stomach. Although intoxicated, he was able to state what he had done, and where the phial, which had contained the laudanum, was to be found. He assisted in dressing himself, and, by the aid of a friend, walked into an adjoining room.

One scruple of sulphate of zinc, dissolved in half a pint of warm water, was administered, which, in the course of ten minutes was repeated, irritating the fauces with a feather. The patient was directed to walk up and down the room between two persons, and was by no means particularly drowsy. A large portion of citric acid, dissolved in warm water, was repeatedly given; and, besides the two scruples of sulphate of zinc, ten grains of sulphate of copper were given, in two doses; as also, in two doses, twenty-four grains of tartrate of antimony, all dissolved in copious draughts of warm water, irritating the fauces with a probang; but without exciting vomiting. On asking the patient several times whether he felt sick, he as often replied, "Never was sick in my life." Meanwhile he became quite sober, and related where he had bought the drug,—how long he had had it,—what conversation passed between himself and the druggist. He also entered into conversation respecting his worldly affairs,—conversed with shrewdness and humour,—and, finally, after a lapse of five hours, would not allow persons to assist him in walking about the room, as "he could do so himself;" adding, "there was nothing the matter with him."

Conceiving that Mr. W. might have wasted the greater part of the laudanum while conveying it to his lips, or purposely thrown part of it away, and finding him apparently unaffected by opium, or indeed any thing else, from eleven o'clock P.M. to four A.M., I left him.

A little before nine o'clock, I was sent for, when the patient was very drowsy; his breathing was slightly affected; his skin cold; his face of a purple hue; and pulse rather feeble. As so many attempts at exciting and provoking vomiting had failed, little or no advantage could be expected from them now that the opium had remained ten hours in the system. Cold water was at this time frequently dashed upon the head. Citric acid, in warm water, was again frequently given; as also mustard, sharpened with acetic acid and mixed with warm water; but without exciting the energy of the nervous system.

By this time a great number of persons had collected in front of Mr. W.'s house, and the house itself became unavoidably so thronged, as to make the case one of great public notoriety; I therefore requested that every medical practitioner in the town might be sent for, three of whom (all who were in Frome at the moment,) came. We administered three or four strong aloetic lavements, without producing any alvine discharge. Cloths dipped into boiling water were applied to the abdomen and back, instead of common vesicatories; which, with urtication, were scarcely felt by the patient, notwithstanding he had sufficient control over the voluntary powers to void urine, at his own desire. Shortly after this, the pulse became much more feeble and indistinct; the skin quite cold; the breathing less perceptible; the speech and the powers of mind gradually vanished; and, finally, he died like one in a trance:—he fell, without an effort, into the sleep of death!

In the case of Mr. J. B. (see vol. xxxi.) which was treated successfully, the two first doses of sulphate of zinc, as given in the above instance, produced copious vomiting; and what remained of the laudanum in the system was counteracted by the foregoing stimuli and vegetable acids, although I found him in a state of insensibility, and almost in *articulo mortis, three hours after the poison had been swallowed.*

In those few cases of poisoning by opium which I have seen, the patients laboured under its narcotic and sedative influence in two or three hours after it had been taken into the stomach; but, in the case of Mr. J. W., such was the effect of the free and long-continued use of tobacco, of ardent spirits, and of fermenting liquors;—such, also, was the effect of taking opium while in a state of inebriety, diluted and counteracted, as it must have been, by those plentiful potations of strong beer, in which he had just indulged, together with strong emetics, citric acid, and warm water, subsequently given,—that nearly two ounces of laudanum were incapable of producing a narcotic and sedative influence upon the patient till *ten hours* after the poison had been in the system, except in increasing that insusceptibility of the stomach to the action of emetics, which was in a peculiar and extraordinary manner *constitutional,* and the effect of intemperance and the habitual use of tobacco.

The foregoing case occurred on the 12th of July last; and I have to regret that Mr. Jukes's highly interesting experiments, instituted with a view to evacuate, by mechanical agency, laudanum from the stomach, were not known to me, although some of them were published, as he states, on the 1st of that month.

It appears, from Mr. Jukes's valuable communication in the Journal for November, that he had suggested the use of the above instrument, as "a new means of extracting opium, &c. from the stomach, in several past Numbers of a periodical publication of the present year:" he is not, however, entitled to the full and exclusive merit of having been the first to suggest the use of such an instrument; for Orfila had described a similar method of evacuating the stomach, in the first volume of his "Toxicology:" but, what is far more creditable and laudable, he has been the first to *prove* its efficiency to perform the office required, and to establish confidence in its use, by evacuating with it the contents of the stomach (in which large quantities of opium were suspended,) of several dogs,—of three persons,—and even of himself!

*Frome, Somerset; Jan. 10th,* 1823.

